# Apolipoprotein E ε3/ε4 and coronary artery disease: lipid and non-lipid mechanisms in personalized prevention — narrative review

**DOI:** 10.3389/fcvm.2026.1885898

**Published:** 2026-09-18

**Authors:** Rodica Diaconu, Vlad Pădureanu, Răzvan Ilie Radu, Maria Iovănescu, Oana Mirea, Diana Ruxandra Hădăreanu, Ionuţ Donoiu

**Affiliations:** 1 CardioEcho Clinic, Craiova, Romania; 2 Department of Internal Medicine, University of Medicine and Pharmacy of Craiova, Craiova, Romania; 3 Intensive Cardiac Care Unit, Emergency Institute for Cardiovascular Diseases “C. C. Iliescu”, Bucharest, Romania; 4 Department of Cardiology, University of Medicine and Pharmacy of Craiova, Craiova, Romania; 5 Department of Cardiology, University of Medicine and Pharmacy Carol Davila, Bucharest, Romania

**Keywords:** APOE ε3/ε4, apolipoprotein E, atherosclerosis, cardiovascular genetics, coronary artery disease, inflammation, lipid metabolism, personalized prevention

## Abstract

**Background:**

Coronary artery disease (CAD) remains a leading cause of cardiovascular morbidity and mortality worldwide. Although traditional risk factors—including dyslipidemia, hypertension, diabetes mellitus, obesity, and smoking—account for a substantial proportion of CAD risk, they do not fully explain interindividual differences in disease onset, progression, or severity. Apolipoprotein E (ApoE) is a multifunctional protein involved in lipoprotein metabolism, inflammatory regulation, oxidative balance, endothelial function, and tissue repair. The APOE gene harbors three major alleles—*ε*2, *ε*3, and *ε*4—generating six common genotypes. Among these, the *ε*3/ε4 genotype has been associated in several studies with an unfavorable lipid profile and increased susceptibility to CAD and premature CAD.

**Objective:**

This narrative review synthesizes current evidence on the association between the APOE *ε*3/ε4 genotype and CAD, with emphasis on lipid-mediated and non-lipid biological mechanisms, interactions with conventional cardiovascular risk factors, and potential implications for personalized cardiovascular prevention. Throughout the review, evidence obtained directly in ε3/ε4 carriers is distinguished from evidence extrapolated from ε4-carrier, ε4/ε4, or overall APOE-polymorphism analyses.

**Methods:**

A narrative literature search was conducted in PubMed, Embase, Scopus, and Web of Science from database inception to August 2026, using the terms “Apolipoprotein E,” “APOE ε3/ε4,” “APOE polymorphism,” “coronary artery disease,” “premature coronary artery disease,” “lipid metabolism,” “atherosclerosis,” and “cardiovascular genetics.” English-language original articles, narrative reviews, and meta-analyses relevant to the APOE ε3/ε4 genotype and cardiovascular disease were included. Retrieved studies were classified by genotype contrast and by level of evidence (human observational, human interventional, or experimental).

**Summary:**

ApoE plays a central role in plasma lipoprotein transport and hepatic clearance of triglyceride-rich lipoprotein remnants. The *ε*4 isoform has been associated with higher LDL cholesterol, apolipoprotein B, and remnant-lipoprotein concentrations, which may partly explain its association with coronary atherosclerosis. Beyond lipid transport, ApoE modulates macrophage activity, inflammatory signaling, oxidative stress, endothelial function, apoptosis, angiogenesis, and tissue repair. These non-lipid pathways derive predominantly from *in vitro* and animal models and remain unvalidated in human ε3/ε4 carriers. Where adjusted, ε3/ε3-referenced estimates are available, the effect size is modest, with odds ratios of approximately 1.5 for unselected CAD and 1.5–2.2 for premature coronary phenotypes.

**Conclusions:**

The APOE *ε*3/ε4 genotype may represent a genetic modifier of CAD risk, particularly when combined with traditional cardiovascular risk factors. Current evidence remains heterogeneous, and APOE genotyping should be regarded as a potential adjunctive marker and not a stand-alone risk prediction tool; this is distinct from its established diagnostic role in dysbetalipoproteinemia. Prospective studies integrating genetic, lipid, inflammatory, and imaging data are needed to clarify its incremental clinical value beyond LDL-C, apolipoprotein B, lipoprotein(a), and coronary artery calcium.

## 1 Introduction

Coronary artery disease is a complex, multifactorial disorder resulting from the interaction of genetic susceptibility, traditional cardiovascular risk factors, environmental exposures, and inflammatory and metabolic pathways. It remains one of the leading contributors to global morbidity and mortality, accounting for over seven million deaths annually despite substantial advances in prevention, diagnosis, and treatment (1–3). Age, sex, hypertension, diabetes mellitus, smoking, obesity, and dyslipidemia remain central to cardiovascular risk stratification, but they do not fully explain interindividual variability in CAD onset, severity, or clinical outcomes (4–6). This discrepancy has intensified interest in genetic markers that may refine risk prediction, particularly in patients with premature disease, a strong family history, or multiple metabolic comorbidities.

**Table 1:** Summary of major APOE isoforms and their cardiovascular relevance.

Isoform/Allele	Typical Lipid Tendency	Potential Cardiovascular Relevance	Selected Ref.
ε2 (ApoE2)	Lower LDL-C; higher TG in some settings; lowest LDL receptor-binding affinity (<2% of ApoE3)	Often considered relatively cardioprotective for LDL-mediated atherosclerosis; risk of type III hyperlipoproteinemia in ε2/ε2 homozygotes with metabolic cofactors	(25,31)
ε3 (ApoE3)	Reference variant; intermediate LDL-C and TG levels	Baseline or intermediate cardiovascular risk; most common isoform worldwide; conventional comparator genotype	(12,16)
ε4 (ApoE4)	Higher LDL-C, total cholesterol and ApoB in most cohorts; lower circulating ApoE concentrations	Associated with CAD, premature CAD, and greater atherosclerosis severity in multiple cohorts; non-lipid effects on inflammation, oxidative stress, macrophage function and endothelial repair are experimentally supported but not validated in human ε3/ε4 carriers (Table 3)	(17,18,30,35–37,41–46)

Apolipoprotein E is among the most extensively studied proteins in lipoprotein metabolism. Initially characterized for its role in cholesterol transport and hepatic lipoprotein clearance, ApoE is now recognized as a multifunctional molecule involved in lipid homeostasis, immune modulation, oxidative stress regulation, endothelial function, and tissue repair (7, 8). It is a 34-kDa protein and an important structural component of very-low-density lipoproteins (VLDL), chylomicron remnants, and high-density lipoproteins (HDL). ApoE is synthesized predominantly in the liver but is also produced by the brain, kidney, adipocytes, smooth muscle cells, and macrophages (9–11).

The APOE gene is polymorphic, with three major alleles—ε2, ε3, and ε4—determined by two single-nucleotide polymorphisms (SNPs), rs429358 and rs7412, at positions encoding amino acids 112 and 158 of the mature protein (12–15). These alleles generate six common genotypes: ε2/ε2, ε2/ε3, ε2/ε4, ε3/ε3, ε3/ε4, and ε4/ε4. The ε3 allele is the most prevalent isoform, occurring in approximately 62% of the population, and is generally regarded as the reference variant (16). Allelic differences influence receptor-binding affinity, lipoprotein clearance, and downstream lipid metabolism, which may translate into differential patterns of cardiovascular susceptibility (Table 1).

The potential relationship between APOE polymorphisms and CAD has been examined in diverse populations, with inconsistent results. Several studies support an association between the ε4 allele—particularly the ε3/ε4 genotype—and increased CAD risk, premature CAD, and more severe coronary atherosclerosis (17, 18). Other studies have not confirmed APOE as an independent CAD risk factor (19). The most plausible explanation is not that the association is absent, but that its effect size is small and highly context-dependent. APOE ε3/ε4 may therefore be better interpreted as a modifier of risk exposure rather than a deterministic CAD genotype.

The terms “ε4 allele,” “*ε*4 carrier,” and “APOE polymorphism” are often used interchangeably, although they denote different exposures: ε4-carrier analyses pool ε2/ε4, ε3/ε4, and ε4/ε4 individuals with differing phenotypes, and allele-level analyses do not permit genotype-specific inference. Evidence obtained with ε3/ε4 as a defined exposure group, ideally against an ε3/ε3 comparator, is therefore reported separately below and indicated for each study in Table 2.

**Table 2:** Key clinical studies on the association between the APOE ε3/ε4 genotype or ε4 allele and coronary artery disease. Expanded to report genotype contrast, outcome definition, adjustment, effect estimates, and whether the evidence is ε3/ε4-specific.

Author, Year	Population (n)	Design	Genotype contrast (comparator)	CAD/PCAD definition	Adjustment	Effect estimate (95% CI)	ε3/ε4-specific?	Ref.
Zeng et al., 2026	Han Chinese, mid/high altitude (628 CAD/628 controls)	Case-control	ε3/ε4 vs. ε3/ε3	≥ 50% stenosis of a main coronary artery or major branch, or CAD hospitalisation	Sex, age, altitude, smoking, alcohol, hypertension, diabetes (not lipids)	aOR 1.51 (1.09–2.11), *p* = 0.014; ε4/ε4 aOR 2.73 (0.74–10.09), NS	Yes — direct	(30)
Wang et al., 2026	Southern Chinese (379 PMI/628 controls)	Case-control	ε3/ε4 vs. ε3/ε3	Premature MI: onset ≤ 55 y men, ≤ 65 y women (4th UDMI criteria)	Smoking, hypertension, dyslipidemia, alcohol, diabetes	aOR 1.55 (1.04–2.31), *p* = 0.032; ε2/ε3 protective (7.7% vs. 18.8%)	Yes — direct	(39)
Li et al., 2024	Southern Chinese Hakka (301 PCAD/402 controls)	Case-control	ε3/ε4 vs. ε3/ε3	PCAD: onset ≤ 55 y men, ≤ 65 y women	BMI, smoking, alcohol	aOR 2.20 (1.36–3.56), *p* = 0.001	Yes — direct	(38)
Wei et al., 2024	Chinese hypertensive patients	Case-control	ε3/ε4 vs. ε3/ε3	Coronary atherosclerosis in hypertension	Conventional risk factors	Independent association reported	Yes — direct	(40)
Shao et al., 2022	Multi-ethnic meta-analysis	Meta-analysis	Allele level (ε2, ε3, ε4)	Myocardial infarction	Varies by included study	ε4 associated with increased MI risk; ε2 protective	No — allele level	(55)
Schmitz et al., 2007	German (Aachen study)	Case-control	ε4 allele	Premature myocardial infarction	Stratified by hypercholesterolaemia	ε4 association strongest in patients WITHOUT hypercholesterolaemia	No — allele level	(54)
Chen et al., 2024	Chinese T2DM patients	Case-control	ε3/ε4 vs. other genotypes	T2DM complicated by CAD	Reported as independent association; full covariate set not specified	Significant independent association; point estimate not extractable	Yes — direct	(35)
Liu & Wu, 2021	Southern Chinese Hakka (1,129 CAD/1,014 controls)	Case-control	ε3/ε4 vs. remaining genotypes	Clinically diagnosed CAD	Unadjusted genotype comparison	*χ*^2^ = 8.08, *p* = 0.005; no OR reported	Yes — direct (unadjusted)	(36)
Bos et al., 2021	UK Biobank, white European (345,659)	Prospective cohort	ε3/ε4 vs. ε3/ε3	Incident CAD via hospital admission linkage	Age, sex; lifestyle interaction terms tested	HR range ≈ 0.97–1.15; no significant gene–lifestyle interaction	Yes — direct	(49)
Abd El-Aziz & Mohamed, 2016	Egyptian (*n* = 200)	Case-control	ε4 carriers (ε3/ε4 + ε4/ε4)	PCAD: onset < 45 y men, < 55 y women	Classical risk factors considered alongside genotype	Significant association; genotype-specific OR not reported	No — ε4 carriers pooled	(17)
Zhao et al., 2017	Multi-ethnic meta-analysis	Meta-analysis	Allele level (ε2, ε3, ε4)	PCAD; age thresholds varied across included studies	Varies by included study	Significant overall; ε2 protective in Caucasians, risk factor in Asians; substantial heterogeneity	No — allele level	(18)
Karjalainen et al., 2019	Finnish adults	Prospective cohort	ε4 allele	Incident CAD	Ceramide levels examined as mediators	Significant; ceramides proposed as mediators	No — allele level	(41)
Balcerzyk et al., 2007	Polish PCAD patients	Case-control	ε4 allele	Premature ischaemic heart disease	Stratified by smoking and hypercholesterolaemia; no formal interaction test	Joint effects reported; departure from additivity not tested	No — allele level	(44)
Luo et al., 2017	Chinese T2DM patients	Case-control	ε2/ε3/ε4 genotypes	CAD in T2DM	Not fully specified	Significant association reported	Partial	(45)
Karahan et al., 2015	Turkish AMI patients	Case-control	ε3/ε4 + ε4/ε4 combined	CAD severity (Gensini score)	Not fully specified	Significant association with severity	No — ε4 genotypes pooled	(37)
Long et al., 2019	Chinese patients	Case-control	ε2/ε3/ε4 + MTHFR	CAD risk and severity	Not fully specified	Significant association reported	No	(46)
Wang et al., 1995	Australian, angiographic	Cross-sectional	ε4 carriers	Angiographic extent of coronary atherosclerosis	Limited	Greater angiographic extent in ε4 carriers	No — ε4 carriers pooled	(47)
Petrovic et al., 2000	Slovenian	Case-control	ε2/ε3/ε4	Premature CAD	Lipid parameters considered	Null — not an independent predictor	No	(19)
Pitsavos et al., 2005	Greek familial hypercholesterolaemia	Cross-sectional	ε2/ε3/ε4	CAD in FH	Lipid levels considered	Null — no association	No	(48)

Effect estimates are reproduced only where the source publication reported them; “not extractable” indicates that the study reported statistical significance without a genotype-specific point estimate. aOR, adjusted odds ratio; HR, hazard ratio; NS, not significant; PCAD, premature coronary artery disease; T2DM, type 2 diabetes mellitus; FH, familial hypercholesterolaemia.

Importantly, the effects of ApoE on cardiovascular disease should not be interpreted exclusively through changes in LDL cholesterol or total cholesterol. ApoE participates in macrophage biology, inflammatory regulation, oxidative balance, endothelial function, apoptosis, angiogenesis, and tissue repair (20, 21)—mechanisms directly relevant to plaque initiation, progression, and instability. Few reviews have focused specifically on the ε3/ε4 genotype while integrating lipid-dependent and lipid-independent mechanisms with conventional cardiovascular risk factors. The present review aims to address this gap, while making explicit the level of evidence supporting each proposed mechanism (Table 3).

**Table 3:** Level of evidence supporting each proposed non-lipid mechanism linking ApoE4 to coronary risk.

Proposed mechanism	Nature of supporting evidence	Demonstrated in human ε3/ε4 carriers?	Level of evidence	Ref.
Higher LDL-C/ApoB and impaired remnant clearance	Human observational cohorts with genotype-stratified lipid measurement	Yes — replicated across several ancestries	Established (human)	(26,28–30)
Pro-inflammatory macrophage phenotype	Myeloid-specific isoform-replacement mice showing intrinsic ApoE4 pro-inflammatory effect; murine macrophages; monocyte culture	Minimal — one report of APOE genotype modifying hs-CRP performance; no genotype-stratified human vascular data	Experimental, minimal human	(7,20,56–59)
Reduced macrophage cholesterol efflux/foam-cell formation	Cell-culture demonstration of impaired ApoE4 recycling; ABCA1/ABCG1/LXR pathway characterised independently of APOE genotype	No — efflux capacity never compared across APOE genotypes in a clinical cohort	Experimental only	(60–62)
Reduced antioxidant capacity/oxidative stress	*In vitro* and animal work; human data largely from non-vascular tissues	Partial — systemic markers only, not vascular or genotype-matched for ApoB	Experimental, limited human	(63)
Endothelial dysfunction/impaired repair	Animal and cell models	No — endothelial function not systematically compared by genotype	Experimental only	(63,70)
Impaired efferocytosis/necrotic core expansion	Inference from ApoE–LRP1 interaction; efferocytosis itself established via MerTK, LRP1, CD47–SIRP*α*, none ApoE-isoform-specific	No — apoptotic-cell clearance never measured as a function of ApoE isoform in any model	Hypothesis only	(62,65)
Altered angiogenesis/tissue repair	*In vitro* inhibition of endothelial proliferation by ApoE	No	Experimental only	(64)

## 2 Literature search and selection

A narrative literature search was conducted in PubMed, Embase, Scopus, and Web of Science from database inception to August 2026. The following search terms were used in various combinations with Boolean operators (AND, OR): “Apolipoprotein E,” “APOE ε3/ε4,” “APOE polymorphism,” “coronary artery disease,” “premature coronary artery disease,” “lipid metabolism,” “atherosclerosis,” “cardiovascular genetics,” and “personalized prevention.” Reference lists of retrieved articles were hand-searched for additional relevant publications.

A single strategy was used, combining an exposure block (“Apolipoprotein E”[MeSH] OR “APOE”[tiab] OR “APOE polymorphism”[tiab] OR “APOE e3/e4”[tiab]) with a topic block (“Coronary Artery Disease”[MeSH] OR “premature coronary artery disease”[tiab] OR “Atherosclerosis”[MeSH] OR “Lipid Metabolism”[MeSH] OR “cardiovascular genetics”[tiab] OR “personalized prevention”[tiab]) using the Boolean operator AND, with an English-language filter applied. Equivalent syntax was adapted for Embase (Emtree), Scopus (TITLE-ABS-KEY), and Web of Science (Topic). Full database-specific strings are provided as Supplementary Material.

Inclusion criteria were: (1) English-language publications; (2) original research articles, narrative reviews, and meta-analyses; (3) reporting on associations between APOE genotype—with specific emphasis on the ε3/ε4 genotype—and CAD, premature CAD, lipid metabolism, or ApoE-mediated biological mechanisms. Studies with fewer than five participants, conference abstracts without full text, and publications focused exclusively on APOE in neurodegenerative disease without cardiovascular outcomes were excluded. Records were screened at title and abstract level and potentially relevant full texts were assessed. Selection was purposive, with priority given to studies reporting a defined genotype contrast, to meta-analyses, and to primary experimental sources for mechanistic claims. The final narrative synthesis included 79 references, comprising clinical association studies, meta-analyses, mechanistic experimental studies, and methodological sources. Of these, 18 studies directly addressed CAD, premature CAD, myocardial infarction, or coronary atherosclerosis outcomes in relation to APOE genotype and are summarised in Table 2.

### 2.1 Rationale for a narrative design

This review was conducted as a narrative synthesis. Narrative and systematic reviews serve different purposes and are complementary rather than hierarchically ordered (22); the aim here is to integrate human association data with experimental mechanistic evidence that shares no common effect measure, and pooling would in any case be inappropriate given that the primary studies differ in the exposure itself (allele carriage, ε4-carrier status, or ε3/ε4 genotype), in the comparator, in outcome definition, and in whether lipids were adjusted for. Reporting follows SANRA, the validated instrument for non-systematic reviews (23). No protocol was registered, formal risk-of-bias instruments were not applied, and no quantitative pooling was performed; the resulting susceptibility to selection bias is addressed in Section 10.

## 3 APOE biology and genetic polymorphisms

ApoE belongs to the family of exchangeable amphipathic apolipoproteins and is composed of 299 amino acids (7, 8). Its principal biological function is to mediate the binding of lipoprotein particles to cell-surface receptors—primarily members of the LDL receptor (LDLR) family and LDL receptor-related protein 1 (LRP1)—thereby facilitating the internalization and hepatic clearance of triglyceride-rich lipoprotein remnants and contributing to the redistribution of lipids between tissues (7, 8, 16).

The APOE gene is located on chromosome 19q13.2 and contains four exons and three introns (14). The three major alleles are determined by two SNPs—rs429358 (C112R) and rs7412 (R158C)—which produce amino acid substitutions at positions 112 and 158 of the mature protein (13, 15). The resulting isoforms differ in their cysteine-arginine substitution pattern: ApoE2 (Cys112, Cys158), ApoE3 (Cys112, Arg158), and ApoE4 (Arg112, Arg158). These structural differences confer distinct receptor-binding affinities, lipid binding properties, and lipoprotein metabolic profiles (16).

APOE polymorphism frequencies vary markedly across populations, with the ε3 allele being the most prevalent worldwide, the ε4 allele more common in Northern Europeans and African Americans, and both ε2 and ε4 less frequent in Asian populations (24).

ε3 is the most frequent isoform in all populations studied and is conventionally used as the reference genotype in association studies. ApoE2, with substantially reduced LDL receptor-binding affinity (less than 2% of ApoE3), is generally associated with lower LDL cholesterol levels; however, in the presence of metabolic or genetic cofactors, ε2/ε2 homozygotes may develop type III hyperlipoproteinemia (25). ApoE4 differs structurally from ApoE3 through a domain interaction between Arg61 and Glu255 and has consistently been associated with higher LDL cholesterol and total cholesterol levels, as well as lower circulating ApoE plasma concentrations (25, 26). These lipid effects provide a plausible biological basis for the cardiovascular relevance of the ε3/ε4 genotype.

The ε4 allele is the ancestral APOE variant, from which ε3 and subsequently ε2 arose (27).

## 4 APOE ε3/ε4 and lipid metabolism

ApoE functions as a high-affinity ligand for hepatic LDLR and LRP1, mediating the uptake and clearance of chylomicron remnants and VLDL remnants from the circulation (7, 8, 16). Differences in the receptor-binding capacity of ApoE isoforms directly affect the rate of lipoprotein remnant clearance and downstream plasma lipid concentrations.

Carriers of the ε4 allele typically exhibit higher total cholesterol and LDL cholesterol levels compared with ε3/ε3 homozygotes, whereas ε2 carriers tend to have lower LDL cholesterol but may display elevated triglyceride concentrations (25, 26). In a comprehensive evaluation of ApoE's influence on plasma lipids across multiple studies, ε4 carriers showed consistently higher total cholesterol and LDL cholesterol, while ε2 carriers demonstrated lower total cholesterol and a tendency toward elevated triglycerides (26). Other studies have confirmed higher LDL cholesterol and ApoB concentrations among ε4 carriers, together with significantly lower circulating ApoE levels (28). Two datasets provide a genotype-specific rather than allele-level contrast. In a Senegalese cohort, total cholesterol was significantly higher in individuals with the APOE ε3/ε4 genotype compared with ε3/ε3 homozygotes (29), and in a Han Chinese case-control series LDL-C (2.37 ± 0.89 vs. 2.22 ± 0.79 mmol/L), ApoB (0.79 ± 0.24 vs. 0.74 ± 0.22 g/L) and the ApoB/ApoA-I ratio (0.75 ± 0.23 vs. 0.68 ± 0.23) were all significantly higher in ε3/ε4 than in ε3/ε3 participants, with correspondingly lower HDL-C (all *p* < 0.05) (30), while a large multiethnic elderly cohort further documented lower LDL cholesterol in ε2 carriers relative to other genotypes (31).

Describing this phenotype in terms of LDL-C alone understates it. The causal quantity in atherogenesis is the number of ApoB-containing particles, not their cholesterol content, and the two diverge in insulin resistance, hypertriglyceridemia, and metabolic syndrome (32) — the settings in which ε3/ε4 carriers are reported to be at greatest risk. Since reduced ApoE-mediated clearance causes retention of remnant particles, each carrying one ApoB molecule, ApoB and non-HDL-C describe the ε3/ε4 phenotype more accurately than LDL-C. Accordingly, the ApoB/ApoA-I ratio discriminated ε3/ε4 from ε3/ε3 as clearly as LDL-C did (30), and mass-spectrometric profiling has confirmed that APOE genotype shapes the wider apolipoprotein distribution (33). Lipoprotein(a) contributes to residual atherosclerotic risk (34) but is not known to be modified by APOE genotype (30), and represents an independent axis of risk.

The ε3/ε4 genotype may therefore contribute to CAD risk through a more atherogenic lipid phenotype characterized by elevated ApoB-containing particle number, non-HDL cholesterol, remnant cholesterol, and LDL cholesterol—all recognized mediators of cholesterol deposition within the arterial wall, macrophage foam-cell formation, and plaque growth. However, lipid differences alone do not fully account for the heterogeneity of CAD risk observed across APOE studies, underscoring the importance of the non-lipid mechanisms discussed in Section 7.

## 5 APOE ε3/ε4 and coronary artery disease

The association between APOE polymorphisms and CAD has been examined in diverse populations with varying results (Table 2). The evidence is presented below in three tiers: studies in which ε3/ε4 was a defined exposure group; studies reporting on ε4 carriers or on APOE polymorphism as a whole, from which ε3/ε4-specific conclusions can only be inferred; and null or contradictory findings.

### 5.1 Evidence with ε3/ε4 as a defined exposure group

The most informative estimate available comes from a case-control study of 628 CAD patients and 628 matched controls in a Han Chinese population, using ε3/ε3 as the reference genotype. The ε3/ε4 genotype was more frequent among cases than controls (18.2% vs. 11.9%, *p* = 0.002) and remained an independent predictor of CAD after adjustment for sex, age, smoking, alcohol use, hypertension, and diabetes (adjusted OR 1.51, 95% CI 1.09–2.11, *p* = 0.014). The ε4/ε4 genotype showed a larger crude effect (OR 3.91, 95% CI 1.08–14.11) that lost significance after adjustment (OR 2.73, 95% CI 0.74–10.09), which the authors attributed to its low prevalence and consequent loss of power (30). A modest but statistically robust effect for ε3/ε4, alongside an imprecise estimate for ε4/ε4, is the main quantitative argument for treating ε3/ε4 as the clinically relevant ε4-containing genotype.

Chen et al. reported that the APOE ε3/ε4 genotype was independently associated with an increased risk of type 2 diabetes mellitus complicated by CAD (35), and in the southern Chinese Hakka population the ε3/ε4 genotype differed significantly in frequency between CAD patients and controls (*χ*^2^ = 8.08, *p* = 0.005) (36), although neither study reported an ε3/ε3-referenced adjusted odds ratio. Karahan et al. reported a significant relationship between APOE polymorphism and CAD severity in patients with acute myocardial infarction (37), with ε3/ε4 and ε4/ε4 analysed together rather than separately.

Two further studies extend the genotype-specific evidence to premature phenotypes, both using ε3/ε3 as comparator and defining premature disease as onset ≤ 55 years in men and ≤ 65 years in women. In 301 patients with premature CAD and 402 controls, ε3/ε4 remained associated after adjustment for body mass index, smoking, and alcohol use (OR 2.20, 95% CI 1.36–3.56) (38). In 379 patients with premature myocardial infarction and 628 controls, ε3/ε4 was independently associated after adjustment for smoking, hypertension, and dyslipidemia (OR 1.55, 95% CI 1.04–2.31) (39), while ε2/ε3 was less frequent among cases (7.7% vs. 18.8%, *p* < 0.001), consistent with a protective ε2 effect.

A third study identified ε3/ε4 as an independent correlate of coronary atherosclerosis in hypertensive patients (40), discussed in Section 8.2. Adjusted ε3/ε3-referenced estimates are therefore now available across four coronary phenotypes, with point estimates between 1.5 and 2.2. Most derive from Chinese Hakka or Han populations, so their consistency does not establish generalisability.

### 5.2 Evidence from ε4-carrier or overall-polymorphism analyses

The following findings concern the ε4 allele or APOE polymorphism as a whole; because ε3/ε4 constitutes the large majority of ε4-containing genotypes in every population studied, these results are likely to be substantially driven by ε3/ε4 individuals, but they do not constitute direct genotype-specific evidence. In Finnish adults, APOE polymorphism was linked to a higher risk of CAD, with plasma ceramides proposed as potential mediators of this relationship (41). Studies in Kashmiri (42) and Northwest Chinese (43) populations similarly suggested an association between the ε4 allele and CAD risk. Balcerzyk et al. found that the ε4 allele acted jointly with conventional risk factors—including smoking and hypercholesterolemia—to increase the risk of premature ischemic heart disease (44). Luo et al. found associations between APOE polymorphisms and CAD in patients with type 2 diabetes mellitus (45). Long et al. described an association between APOE genotype and CAD severity in a case-control study that also examined MTHFR polymorphisms (46). An earlier angiographic study by Wang et al. demonstrated that ε4 carriers had a greater extent of coronary atherosclerosis, as assessed by coronary angiography (47).

### 5.3 Null and contradictory findings

However, not all studies confirm APOE as an independent CAD risk factor. Petrovic et al. did not identify APOE polymorphism as an independent predictor of premature CAD in a Slovenian cohort (19), and Pitsavos et al. found no association between APOE genotype, lipid levels, and CAD in Greek patients with familial hypercholesterolemia (48). In the UK Biobank, hazard ratios for CAD in ε3/ε4 relative to ε3/ε3 participants fell within a narrow range around unity (approximately 0.97–1.15), indicating that in a large, population-based European cohort the genotype-specific effect is small enough to be of limited individual predictive value (49). Similarly, in the study of Zeng et al. the ε4/ε4 genotype was not an independent predictor after adjustment (30). These discordant findings underscore the importance of population-specific genetic backgrounds, environmental exposures, risk factor burden, and differences in study design, sample size, and clinical endpoint definition. Taken together, the quantitative evidence is most consistent with a genotype-specific odds ratio in the region of 1.0–1.5 for ε3/ε4 versus ε3/ε3, with the upper end observed in selected non-European populations and in metabolically high-risk subgroups.

## 6 APOE ε3/ε4 and premature coronary artery disease

Premature coronary artery disease (PCAD)—generally defined as CAD diagnosed before the age of 45–55 years in men and 55–65 years in women—is a particularly relevant phenotype for studying genetic susceptibility, as disease occurs before cumulative lifetime exposure to traditional risk factors can fully account for the atherosclerotic burden (50–52). These thresholds are not standardised, and the variation matters: a cut-off of 45 years in men selects a more genetically enriched population than one of 55 years. The definition applied in each study is reported in Table 2.

Abd El-Aziz and Mohamed reported that APOE, LDLR, and ApoB polymorphisms, together with classical risk factors, were significantly associated with premature CAD (17) (PCAD defined as onset < 45 years in men and < 55 years in women). The ε4 allele was proposed to increase PCAD risk, at least in part through higher LDL cholesterol and total cholesterol concentrations. A meta-analysis by Zhao et al., pooling data from multiple studies, found that APOE polymorphisms were significantly associated with PCAD, although the direction and magnitude of association varied by ethnicity—the ε2 allele appeared protective in Caucasians but was reported as a potential risk factor in Asian populations (18). That meta-analysis was conducted at allele rather than genotype level, so it does not provide an ε3/ε4-specific pooled estimate; the between-study heterogeneity reported by the authors was substantial, consistent with genuine effect modification by ancestry rather than with sampling variation alone.

Systematic reviews and meta-analyses of PCAD risk factors consistently identify dyslipidemia, smoking, hypertension, diabetes mellitus, obesity, and family history as the primary determinants (50–53). Genetic susceptibility—including APOE polymorphisms—is recognized as an additional modifier whose clinical impact depends on the broader cardiometabolic context. Registry data from Indian and Iranian cohorts confirm that conventional risk factors predominate in PCAD patients, but also reveal a substantial proportion of individuals without complete classical explanations, suggesting a role for genetic determinants (50, 52).

The heterogeneity of PCAD findings with APOE may reflect differences in age thresholds used to define premature disease, ethnic background, lifestyle factors, sample size, and variable adjustment for confounders. Adjusted genotype-specific estimates for premature disease (OR 2.20 and 1.55) (38, 39) exceed that for unselected CAD (OR 1.51) (30), the direction expected if premature disease is enriched for genetic susceptibility, though the confidence intervals overlap. The Aachen study reported a similar enrichment, with the ε4 association strongest in patients without hypercholesterolaemia (54), suggesting a component of risk not transmitted through LDL cholesterol; allele-level meta-analysis is broadly concordant (55). Equivalent estimates in European, African, and South Asian PCAD populations remain unavailable.

## 7 Non-Lipid mechanisms linking ApoE4 to coronary risk

Although the lipid-related effects of APOE are well established, ApoE also has functions that may influence atherosclerosis independently of plasma cholesterol levels. These non-lipid mechanisms are particularly relevant to explaining why the association between APOE ε3/ε4 and CAD is heterogeneous and why risk appears stronger in individuals with coexisting metabolic and vascular stressors.

A caveat applies throughout this section. Almost all the data summarised below come from ApoE-knockout mice, isoform-replacement mice, or cell-culture systems. These models show that ApoE4 behaves differently from ApoE3, but not that the difference produces vascular pathology in human ε3/ε4 heterozygotes, who express both isoforms and in whom ApoE3 may compensate. No isoform-specific defect has been demonstrated in coronary tissue from genotyped carriers. The mechanisms below are experimentally supported but clinically unvalidated, and are graded in Table 3.

### 7.1 Inflammation

Inflammation is central to all stages of atherosclerosis, from initial endothelial activation and monocyte recruitment through plaque progression and rupture (2). ApoE has well-characterized immunomodulatory properties; experimental and clinical evidence indicate that ApoE isoforms differ in their inflammatory effects (7, 20), and ApoE has been shown to restrain vascular inflammation partly through microRNA-dependent control of macrophage activation in murine models (56). ApoE4 has been associated with a more proinflammatory phenotype in several biological contexts, including enhanced macrophage inflammatory signaling, greater pro-inflammatory cytokine production, and reduced anti-inflammatory responses compared with ApoE3. The most direct experimental support comes from myeloid-specific isoform-replacement models, in which macrophage-derived ApoE4—and, separately, ApoE2—promoted atherosclerosis through distinct pro-inflammatory mechanisms relative to ApoE3, establishing that the effect is intrinsic to the isoform expressed by the myeloid compartment rather than secondary to circulating lipid differences (57, 58). In the vascular wall, augmented inflammatory signaling may promote endothelial dysfunction, macrophage infiltration, foam-cell formation, and ultimately plaque destabilization. Human data remain sparse and indirect: an early study found that the diagnostic performance of high-sensitivity CRP for three-vessel CAD was modified by APOE genotype (59), but whether circulating inflammatory biomarkers differ between ε3/ε4 and ε3/ε3 individuals independently of ApoB has not been established in an adequately powered cohort.

### 7.2 Macrophage lipid handling and foam-cell formation

Macrophages are central to atherogenesis. After entering the arterial intima, they internalize modified lipoproteins and transform into foam cells, contributing to fatty-streak formation and early plaque development (21). ApoE is produced by macrophages and participates in cholesterol efflux, lipid redistribution, and local regulation of lipoprotein metabolism (60). Sterol loading increases ApoE synthesis and secretion by macrophage-derived foam cells, while pro-inflammatory cytokines may negatively regulate macrophage ApoE production (60).

ApoE is one component of a larger cholesterol-export machinery. Cholesterol loading generates oxysterols that activate the liver X receptors (LXRα/β), which upregulate ABCA1, ABCG1, and APOE itself. ABCA1 exports free cholesterol and phospholipid to lipid-poor apolipoprotein A-I, while ABCG1 exports cholesterol to mature HDL; combined deficiency produces massive foam-cell accumulation in mice (61). Macrophage-derived ApoE acts alongside them as a secreted cholesterol acceptor and is itself an LXR target gene (61), so its role appears contributory and partly redundant. ABCA1 loss-of-function in humans (Tangier disease) causes premature coronary atherosclerosis; no comparable phenotype has been attributed to isoform-specific ApoE dysfunction.

A dysfunctional ApoE4 isoform may therefore impair macrophage lipid handling, reduce cholesterol efflux efficiency, and promote intracellular lipid accumulation within the arterial wall. The most direct support is the impaired intracellular recycling of ApoE4 relative to ApoE3, with consequent cholesterol accumulation in transfected cells (62). Whether this reduces efflux capacity in human ε3/ε4 macrophages has not been tested; serum cholesterol efflux capacity is measurable and predicts events, but has never been compared across APOE genotypes in a clinical cohort.

### 7.3 Oxidative stress and endothelial dysfunction

Oxidative stress contributes to LDL oxidation, endothelial dysfunction, vascular inflammation, and plaque instability. ApoE has been implicated in antioxidant responses and endothelial protection, and evidence suggests that ε4 carriers may have less effective antioxidant defenses, predisposing to greater oxidative imbalance (63). *In vitro* and animal work indicates that the ApoE isoforms differ in antioxidant capacity in the order ApoE2 > ApoE3 > ApoE4, and that ApoE4 is associated with higher systemic oxidative-stress markers and a lower anti-inflammatory capacity; these differences have been characterised principally in non-vascular tissues, and cardiovascular-specific human data remain limited (63). The ε4 isoform may favor reduced nitric oxide bioavailability and impaired endothelial repair, although flow-mediated dilatation and other endothelial function measures have not been systematically compared between ε3/ε4 and ε3/ε3 individuals. Endothelial dysfunction is an early and pivotal event in atherogenesis and is further worsened by hypertension, smoking, diabetes mellitus, and dyslipidemia. In ε3/ε4 carriers, the coexistence of these stressors may create a biological environment in which the adverse vascular effects of ApoE4 become more clinically manifest.

### 7.4 Apoptosis, angiogenesis, and tissue repair

ApoE has been implicated in apoptosis, angiogenesis, and tissue repair (20, 62, 64). Impaired intracellular recycling of ApoE4—compared with ApoE3—has been associated with intracellular cholesterol accumulation and altered cellular lipid homeostasis (62). ApoE also inhibits endothelial and tumor cell proliferation and modulates angiogenic responses (64), effects that may be relevant to plaque remodeling and neovascularization. In atherosclerotic plaques, defective clearance of apoptotic cells (efferocytosis) contributes to necrotic core expansion and plaque vulnerability (65). The experimentally established determinants of efferocytosis in atherosclerosis are the receptor–ligand systems MerTK, LRP1, and CD47–SIRP*α*. Deletion of Mertk or of macrophage Lrp1, or upregulation of the “don't eat me” signal CD47, each produces accumulation of uncleared apoptotic cells and enlarged necrotic cores in murine lesions (65). ApoE is not among the established efferocytic ligands or receptors.

The suggestion that ApoE4 impairs efferocytosis is therefore an inference, resting on the observations that ApoE is an LRP1 ligand and that ApoE4 shows altered receptor interaction and trafficking (62). Apoptotic-cell clearance has never been measured as a function of ApoE isoform in any model. It remains a testable hypothesis, graded accordingly in Table 3, and should not be presented as an established pathway.

## 8 Interaction of APOE ε3/ε4 with conventional cardiovascular risk factors

The clinical impact of APOE ε3/ε4 on CAD risk is plausibly modified by coexisting conventional cardiovascular risk factors. This may partly explain why APOE-related CAD risk is observed consistently in some cohorts but not in others. “Synergy” and “amplification” are statistical claims requiring a formal interaction term or departure-from-additivity analysis, and most studies cited below report only stratified estimates or joint effects. Where a formal interaction analysis was performed this is stated; elsewhere we use the language of co-occurrence.

The most rigorous test to date examined interaction between APOE genotype and antioxidative lifestyle factors on incident CAD in 345,659 UK Biobank participants and found no convincing evidence of gene–environment interaction on the multiplicative scale (49). This null result provides important context: the biological rationale is coherent, but the largest formal test available has not confirmed it.

### 8.1 Dyslipidemia

Dyslipidemia is the most direct pathway linking APOE ε3/ε4 to CAD. As discussed in Section 4, ε4 allele carriers frequently exhibit higher LDL cholesterol and total cholesterol levels (25, 26, 28, 29, 31), and ε3/ε4 individuals specifically exhibit higher ApoB and ApoB/ApoA-I ratios than ε3/ε3 individuals (30). Elevated ApoB-containing lipoproteins drive cholesterol deposition within the arterial wall, macrophage foam-cell formation, and plaque growth (32). In ε3/ε4 carriers, early recognition and aggressive treatment of dyslipidemia may be particularly relevant, although current cardiovascular prevention guidelines do not yet recommend routine APOE genotyping as a basis for lipid-lowering decisions.

If the effect of ε3/ε4 is transmitted through ApoB-particle concentration, then ApoB is a mediator, not a confounder. Adjusting for lipids therefore estimates the direct, lipid-independent effect and attenuates the total effect; some studies in Table 2 adjust for lipids and some do not, which is one reason their estimates are not comparable. The residual association after lipid adjustment is the quantity attributable to the non-lipid mechanisms of Section 7, and future analyses should address this through formal mediation analysis.

ApoE genotype may also modulate the response to statin therapy, with some evidence suggesting a modestly reduced lipid-lowering effect in ε4 carriers (66). In the GISSI-Prevenzione population, however, APOE genotype did not significantly modify clinical outcomes in statin-treated patients with established CAD (67), so the pharmacogenetic signal is at present confined to intermediate lipid endpoints rather than events. Observational and interventional studies further indicate that adherence to a Mediterranean diet, particularly in combination with statin treatment, may help reduce cardiovascular risk in this population (67).

### 8.2 Hypertension

Hypertension accelerates atherosclerosis through increased arterial wall stress, endothelial dysfunction, vascular remodeling, and pro-inflammatory activation (68–70). Elevated pulse pressure damages vascular endothelial cells, promotes elastic fiber fatigue, and enhances mechanical shear stress, creating conditions that facilitate lipid deposition and plaque initiation. Prospective cohort data confirm that higher baseline blood pressure is associated with markedly elevated CVD risk, and that even stage 1 hypertension, as defined by the 2017 ACC/AHA guidelines, is independently associated with subclinical coronary atherosclerosis (68, 69). In ε3/ε4 carriers, hypertension is a particularly relevant co-exposure. In hypertensive patients specifically, ε3/ε4 was independently associated with coronary atherosclerosis (40), and in the premature myocardial infarction series hypertension carried the largest single effect (OR 4.47, 95% CI 3.27–6.12) while ε3/ε4 remained independently associated after adjustment for it (39). The genotype effect is therefore not simply a proxy for hypertension. Neither study tested interaction formally, so the two are best described as independent, additive contributors.

### 8.3 Smoking

Smoking promotes endothelial injury, inflammation, thrombosis, oxidative stress, and adverse lipid changes through the action of toxic compounds in tobacco smoke (71–73). The relationship between smoking and cardiovascular disease follows a dose-response pattern, and even modest long-term exposure significantly increases CAD risk (71, 72). Balcerzyk et al. documented joint effects of the ε4 allele and conventional risk factors—including smoking and hypercholesterolemia—in the development of premature ischemic heart disease (44), with risk in doubly exposed individuals exceeding that in either exposure alone. Departure from additivity was not formally tested and the analysis was allele-based, so this is consistent with, but does not demonstrate, effect modification. Given the link between tobacco-induced oxidative stress and the antioxidant differences between ApoE isoforms (Section 7.3), it remains a plausible candidate interaction meriting formal testing.

### 8.4 Diabetes mellitus

Diabetes mellitus is associated with accelerated atherosclerosis, endothelial dysfunction, oxidative stress, chronic low-grade inflammation, and atherogenic dyslipidemia. Studies have specifically reported associations between APOE polymorphisms and CAD in patients with type 2 diabetes mellitus (35, 45). The ε3/ε4 genotype may be particularly relevant in diabetic individuals, in whom the combination of hyperglycemia, insulin resistance, dyslipidemia, and chronic inflammation may compound ApoE4-related vascular risk. The finding by Chen et al. that APOE ε3/ε4 is independently associated with CAD in type 2 diabetic patients is one of the few genotype-specific results available (35), although it compares genotypes within a diabetic population rather than testing whether diabetes modifies the APOE–CAD relationship.

### 8.5 Obesity and metabolic syndrome

Obesity contributes to CAD risk through multiple intersecting pathways, including insulin resistance, atherogenic dyslipidemia, hypertension, systemic inflammation, and endothelial dysfunction (74). Higher body mass index has been associated with increased cardiovascular risk in numerous epidemiological and Mendelian randomization studies, although this association shows variability by sex, age, and ethnicity (75, 76). Metabolic syndrome—a clustering of abdominal obesity, insulin resistance, dyslipidemia, and elevated blood pressure—further amplifies cardiovascular risk beyond that of individual components (77). In ε3/ε4 carriers, obesity and metabolic syndrome may intensify lipid disturbances and inflammatory activation. This is also where the LDL-C/ApoB discordance discussed in Section 4 is greatest, so part of any apparent excess risk in obese ε3/ε4 individuals may reflect underestimation of atherogenic particle burden by LDL-C.

## 9 Clinical implications

At present, APOE genotyping should not be considered a stand-alone clinical tool for CAD risk prediction. Traditional risk factors, lipid profile, family history, diabetes status, blood pressure, smoking, and validated risk stratification tools (e.g., the Framingham Risk Score, SCORE2, or pooled cohort equations) remain the foundation of cardiovascular risk assessment. Nevertheless, APOE ε3/ε4 may offer potential adjunctive value in selected contexts.

Two questions are often conflated: whether APOE ε3/ε4 is biologically associated with CAD, and whether measuring it changes clinical decisions. The evidence above supports the first but not the second. Clinical utility requires evidence that genotype adds discrimination or reclassification beyond established predictors, and no such analysis has been published. An odds ratio of approximately 1.5 (30) is well below the magnitude needed to move an individual across a treatment threshold; coronary artery calcium reclassifies a substantial proportion of intermediate-risk individuals, and Lp(a) above 50 mg/dL confers a comparable hazard while being directly measurable and increasingly actionable (34). Any incremental value would also have to be shown over ApoB rather than LDL-C, since much of the genotype effect is expected to be transmitted through ApoB concentration (Section 8.1).

The established uses of APOE genotyping in lipidology are diagnostic, not predictive. Genotyping is indicated in suspected dysbetalipoproteinemia (type III hyperlipoproteinemia), where ε2/ε2 homozygosity with a secondary metabolic factor produces a characteristic remnant-lipoprotein phenotype (14, 25). This indication does not support opportunistic genotyping for CAD risk estimation; the two applications differ in purpose, genotype of interest, and evidential strength.

First, APOE genotyping may help explain interindividual variability in lipid levels and in the response to dietary or pharmacological lipid-lowering interventions, supporting more individualized management decisions. Second, APOE ε3/ε4 may be useful in research settings focused on premature CAD, severe CAD, or CAD occurring in individuals with a strong family history but incomplete explanation by conventional risk factors. Third, APOE may eventually contribute to polygenic or multi-marker cardiovascular risk models, although its incremental predictive value must first be demonstrated prospectively in large, ethnically diverse cohorts.

Critically, the clinical interpretation of APOE ε3/ε4 must remain cautious and contextualized. The presence of the ε3/ε4 genotype does not establish that an individual will develop CAD, and the absence of the ε4 allele does not exclude risk.

## 10 Limitations of current evidence

Several limitations affect the interpretation of current evidence on APOE ε3/ε4 and CAD. First, most published studies are observational in design—cross-sectional, case-control, or retrospective cohort—and therefore cannot establish causality. Second, sample sizes vary substantially across studies, and a number are likely underpowered to detect genotype-specific effects, particularly for the less common ε4/ε4 genotype.

Third, definitions of CAD and premature CAD differ between studies, including variation in age thresholds, angiographic criteria, and clinical endpoints; these are reported per study in Table 2. Endpoint definitions range from ≥ 50% stenosis on angiography to hospital-record-based diagnoses, and the resulting case populations are not equivalent. Fourth, confounder adjustment is inconsistent. Some studies adjust comprehensively for LDL cholesterol, smoking, diabetes mellitus, hypertension, obesity, and medication use, whereas others do not. As set out in Section 8.1, because lipids lie on the causal pathway, adjusting for them changes the estimand from a total to a direct effect; studies that adjust for lipids and those that do not are estimating different quantities.

Fifth, population heterogeneity is substantial. The ε4 allele frequency ranges from roughly 5%–7% in East Asian populations to 15%–20% in Northern Europeans, and the direction of association is inconsistent across ancestries: ε2 was protective in Caucasians but a possible risk factor in Asians (18). Diet is a further modifier, since ApoE mediates dietary lipid handling; the studies reviewed here span dietary patterns from Senegal (29) to Finland (41) to high-altitude western China (30), where altitude and hypoxia have also been proposed as modifiers. These differences cannot be separated with the available data and limit the external validity of any single estimate. Sixth, mechanistic evidence regarding non-lipid pathways largely derives from *in vitro* and experimental animal studies, as detailed in Section 7 and graded in Table 3; their direct contribution to CAD risk in human ε3/ε4 carriers requires prospective clinical validation. Finally, this review is narrative in design and subject to selection bias; a formal systematic review with meta-analysis restricted to studies reporting an ε3/ε4-versus-ε3/ε3 contrast would provide more rigorous and reproducible effect estimates, and is now feasible given the accumulating number of such studies.

## 11 Future perspectives

Future research should focus on several priority areas. Large prospective studies are needed to determine whether APOE ε3/ε4 improves CAD risk prediction beyond traditional risk factors, lipid profile, family history, and established polygenic risk scores, reported as reclassification metrics rather than odds ratios alone. Mendelian randomization studies using large biobanks (e.g., UK Biobank, FinnGen) could help disentangle the causal contribution of APOE ε3/ε4 from the confounding inherent in conventional observational designs, and multivariable Mendelian randomization conditioning on ApoB would directly address whether any lipid-independent effect exists. Studies should also evaluate whether APOE genotype modifies the relationship between LDL cholesterol, ApoB, non-HDL cholesterol, lipoprotein(a), and inflammatory biomarkers with coronary outcomes.

Mechanistic work in humans of known genotype is a further priority, since this is where the evidence base is weakest. Tractable questions include whether serum cholesterol efflux capacity differs between ε3/ε4 and ε3/ε3 individuals matched for ApoB, whether macrophages from ε3/ε4 donors show altered ABCA1/ABCG1 expression or impaired efferocytosis *ex vivo*, and whether markers of oxidative stress and endothelial function differ by genotype independently of lipids. Studies in ε3/ε4 heterozygotes, rather than knockout models, would bridge the experimental and clinical literatures summarised in Tables 2, 3.

An important complementary direction is the integration of APOE genotyping with imaging-based markers of subclinical atherosclerosis. Coronary artery calcium scoring, coronary computed tomography angiography, intravascular ultrasound, and optical coherence tomography may help determine whether ε3/ε4 carriers exhibit greater plaque burden, more vulnerable plaque characteristics, or faster progression of coronary atherosclerosis—findings that would substantially strengthen the case for clinical genotyping in specific high-risk subgroups.

Although evidence from non-coronary cardiovascular phenotypes suggests broader ApoE4 biology (78, 79), such findings remain outside the scope of CAD risk prediction and should not be extrapolated directly to coronary atherosclerosis.

Future research should also evaluate whether APOE genotype modifies the response to lipid-lowering therapy (statins, PCSK9 inhibitors, ezetimibe), lifestyle interventions, or anti-inflammatory strategies. Although current evidence does not support genotype-guided treatment in routine practice, future precision-prevention models may integrate APOE with other genetic, metabolic, imaging, and environmental markers to enable individualized risk stratification and more targeted intervention.

## 12 Conclusions

The APOE ε3/ε4 genotype may contribute to increased susceptibility to coronary artery disease and premature coronary artery disease, particularly in individuals with coexisting traditional cardiovascular risk factors such as dyslipidemia, hypertension, smoking, diabetes mellitus, and obesity. Where adjusted, ε3/ε3-referenced estimates are available, the magnitude of this association is modest: approximately 1.5 for unselected CAD (30), 1.5–2.2 for premature CAD and premature myocardial infarction (38, 39), and close to unity in a large European population cohort (49). Its effects are partly mediated through lipid metabolism—especially higher ApoB-containing particle concentrations and, correspondingly, LDL and non-HDL cholesterol. ApoE also participates in non-lipid pathways relevant to atherosclerosis, including inflammation, oxidative stress, endothelial dysfunction, macrophage lipid handling, and tissue repair, but these are supported by experimental rather than human genotype-specific data and their contribution remains unquantified.

Taken together, the available data suggest that APOE ε3/ε4 is unlikely to be useful as an isolated screening marker. Its value, if confirmed, will probably lie in refining risk estimates in selected groups, particularly patients with premature CAD, diabetes, severe dyslipidemia, or discordance between conventional risk scores and clinical phenotype. Future studies should test whether APOE ε3/ε4 adds measurable predictive value beyond ApoB, Lp(a), diabetes status, smoking, and imaging markers such as coronary calcium or coronary CT angiography, and should report that value as reclassification, not as association.

## Figures and Tables

**Figure 1:**
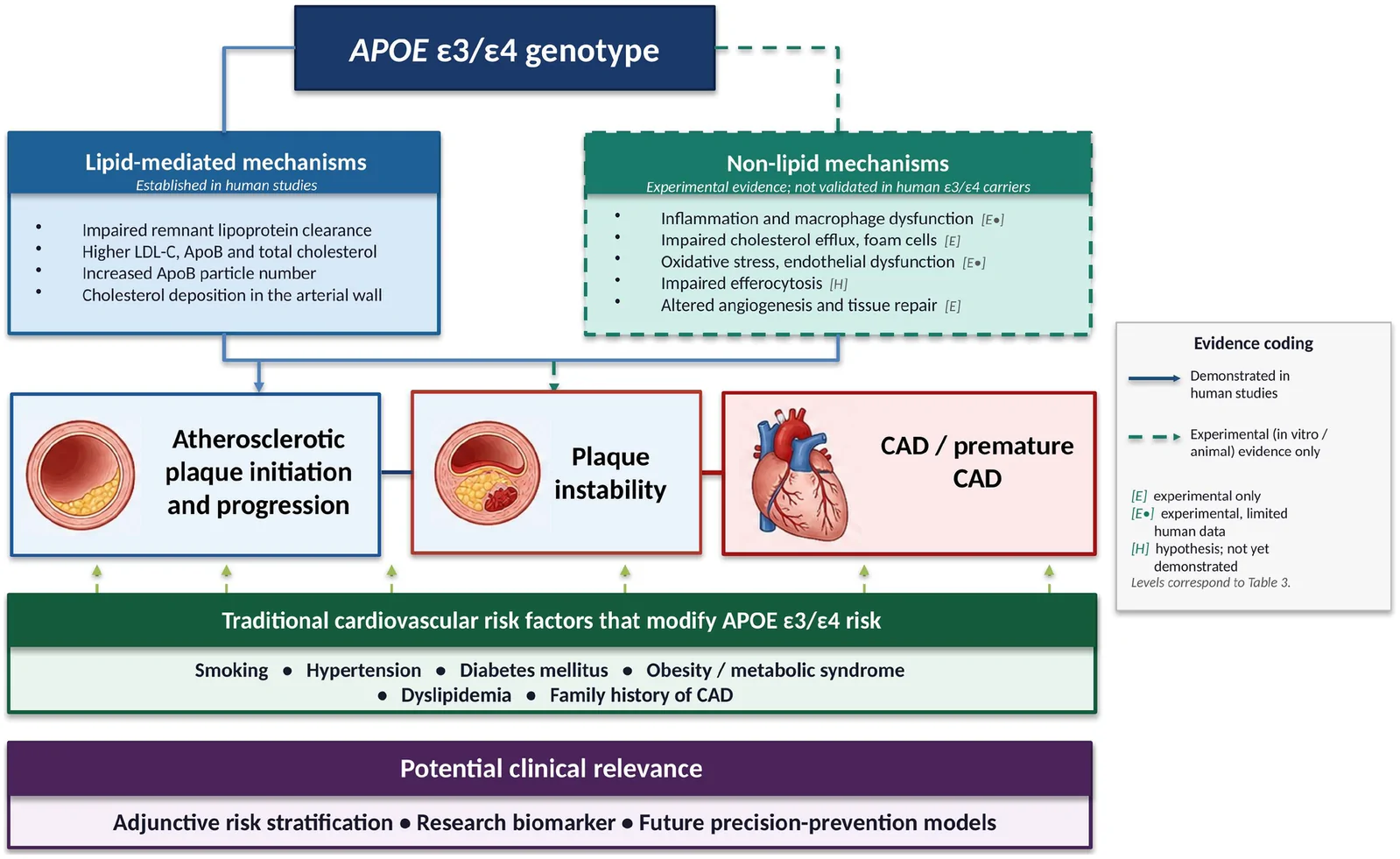
Proposed mechanisms linking the APOE ε3/ε4 genotype to coronary artery disease. Solid arrows denote associations demonstrated in human studies; dashed arrows denote mechanisms supported by experimental (*in vitro* or animal) evidence only and not validated in human ε3/ε4 carriers. Evidence levels correspond to those graded in Table 3.

## References

[1] Gaidai O Cao Y Loginov S. Global cardiovascular diseases death rate prediction. Curr Probl Cardiol. (2023) 48(5):101622. 10.1016/j.cpcardiol.2023.101622 36724816

[2] Stone PH Libby P Boden WE. Fundamental pathobiology of coronary atherosclerosis and clinical implications for chronic ischemic heart disease management—the plaque hypothesis: a narrative review. JAMA Cardiol. (2023) 8(2):192–201. 10.1001/jamacardio.2022.3926 36515941 PMC11016334

[3] Shaya GE Leucker TM Jones SR Martin SS Toth PP. Coronary heart disease risk: low-density lipoprotein and beyond. Trends Cardiovasc Med. (2022) 32(4):181–94. 10.1016/j.tcm.2021.04.002 33872757

[4] Andersson C Johnson AD Benjamin EJ Levy D Vasan RS. 70-year Legacy of the framingham heart study. Nat Rev Cardiol. (2019) 16(11):687–98. 10.1038/s41569-019-0202-5 31065045

[5] Flora GD Nayak MK. A brief review of cardiovascular diseases, associated risk factors and current treatment regimes. Curr Pharm Des. (2019) 25(38):4063–84. 10.2174/1381612825666190925163827 31553287 PMC12994374

[6] Sarebanhassanabadi M Mirjalili SR Marques-Vidal P Kraemer A Namayandeh SM. Coronary artery disease incidence, risk factors, awareness, and medication utilization in a 10-year cohort study. BMC Cardiovasc Disord. (2024) 24(1):101. 10.1186/s12872-024-03769-3 38347457 PMC10863377

[7] Mahley RW. Apolipoprotein E: cholesterol transport protein with expanding role in cell biology. Science. (1988) 240:622–30. 10.1126/science.3283935 3283935

[8] Marais AD. Apolipoprotein E in lipoprotein metabolism, health and cardiovascular disease. Pathology. (2019) 51(2):165–76. 10.1016/j.pathol.2018.11.002 30598326

[9] Kraft HG Menzel HJ Hoppichler F Vogel W Utermann G. Changes of genetic apolipoprotein phenotypes caused by liver transplantation. Implications for apolipoprotein synthesis. J Clin Invest. (1989) 83:137–42. 10.1172/JCI113849 2521341 PMC303653

[10] Driscoll DM Getz GS. Extrahepatic synthesis of apolipoprotein E. J Lipid Res. (1984) 25:1368–79. 10.1016/S0022-2275(20)34454-0 6530593

[11] Zechner R Moser R Newman TC Fried SK Breslow JL. Apolipoprotein E gene expression in mouse 3T3-L1 adipocytes and human adipose tissue and its regulation by differentiation and lipid content. J Biol Chem. (1991) 266:10583–8. 10.1016/S0021-9258(18)99263-X 1709937

[12] Zannis VI Breslow JL Utermann G Mahley RW Weisgraber KH Havel RJ. Proposed nomenclature of apoE isoproteins, apoE genotypes, and phenotypes. J Lipid Res. (1982) 23:911–4. 10.1016/S0022-2275(20)38094-9 7130859

[13] Seripa D D'Onofrio G Panza F Cascavilla L Masullo C Pilotto A. The genetics of the human APOE polymorphism. Rejuvenation Res. (2011) 14(5):491–500. 10.1089/rej.2011.1169 21958003

[14] Khalil YA Rabes JP Boileau C Varret M. APOE gene variants in primary dyslipidemia. Atherosclerosis. (2021) 328:11–22. 10.1016/j.atherosclerosis.2021.05.007 34058468

[15] Lan X Wang Z Zeng Z Yao H Xu W Zhang Y. Association of different combinations of ALDH2 rs671, APOE rs429358, rs7412 polymorphisms with hypertension in middle-aged and elderly people: a case-control study. Int J Gen Med. (2023) 16:915–27. 10.2147/IJGM.S402437 36938306 PMC10017832

[16] Phillips MC. Apolipoprotein E isoforms and lipoprotein metabolism. IUBMB Life. (2014) 66(9):616–23. 10.1002/iub.1314 25328986

[17] Abd El-Aziz TA Mohamed RH. LDLR, ApoB and ApoE genes polymorphisms and classical risk factors in premature coronary artery disease. Gene. (2016) 590(2):263–9. 10.1016/j.gene.2016.05.032 27236033

[18] Zhao QR Lei YY Li J Jiang N Shi JP. Association between apolipoprotein E polymorphisms and premature coronary artery disease: a meta-analysis. Clin Chem Lab Med. (2017) 55(2):284–98. 10.1515/cclm-2016-0145 27394044

[19] Petrovic D Zorc M Peterlin B. Effect of apolipoprotein E polymorphism and apolipoprotein A-1 gene promoter polymorphism on lipid parameters and premature coronary artery disease. Folia Biol (Praha). (2000) 46(5):181–5. 10.14712/fb2000046050181 11055796

[20] Liehn EA Ponomariov V Diaconu R Streata I Ioana M Crespo-Avilan GE. Apolipoprotein E in cardiovascular diseases: novel aspects of an old-fashioned enigma. Arch Med Res. (2018) 49(8):522–9. 10.1016/j.arcmed.2018.08.008 30213474

[21] Davignon J Gregg RE Sing CF. Apolipoprotein E polymorphism and atherosclerosis. Arteriosclerosis. (1988) 8:1–21. 10.1161/01.ATV.8.1.1 3277611

[22] Greenhalgh T Thorne S Malterud K. Time to challenge the spurious hierarchy of systematic over narrative reviews? Eur J Clin Invest. (2018) 48(6):e12931. 10.1111/eci.12931 29578574 PMC6001568

[23] Baethge C Goldbeck-Wood S Mertens S. SANRA—a scale for the quality assessment of narrative review articles. Res Integr Peer Rev. (2019) 4:5. 10.1186/s41073-019-0064-8 30962953 PMC6434870

[24] Diaconu R Dumitrașcu A-R Liehn E Pîrvu A Ioana M Alexandru D. Apolipoprotein E polymorphism in a Romanian population. Rom J Cardiol. (2023) 33(1):19–24. 10.2478/rjc-2022-0031

[25] Mahley RW Rall SC. Apolipoprotein E: far more than a lipid transport protein. Annu Rev Genomics Hum Genet. (2000) 1:507–37. 10.1146/annurev.genom.1.1.507 11701639

[26] Chaudhary R Likidlilid A Peerapatdit T Tresukosol D Srisuma S Ratanamaneechat S. Apolipoprotein E gene polymorphism: effects on plasma lipids and risk of type 2 diabetes and coronary artery disease. Cardiovasc Diabetol. (2012) 11:36. 10.1186/1475-2840-11-36 22520940 PMC3372424

[27] Corbo RM Scacchi R. Apolipoprotein E (APOE) allele distribution in the world. Is APOE*4 a “thrifty” Allele? Ann Hum Genet. (1999) 63(4):301–10. 10.1046/j.1469-1809.1999.6340301.x 10738542

[28] Wang Y Yang S Zhang S Lu X Ma W. Apolipoprotein E gene polymorphism effects on lipid metabolism and risk of cerebral infarction in northwest Han Chinese population. Pharmgenomics Pers Med. (2023) 16:303–12. 10.2147/PGPM.S404663 37051559 PMC10083142

[29] Touré M Diouf NN Thiam S Diop JP Coly MS Mbengue A. Frequencies and distribution of APOE gene polymorphisms and its association with lipid parameters in the Senegalese population. Cureus. (2022) 14(4):e24063. 10.7759/cureus.24063 PMC9097468 35573533

[30] Zeng F Zhang X Zhang M Liu H Li Y Ye F. Association of apolipoprotein E gene polymorphisms with risk of coronary artery disease in a Han Chinese population at middle and high altitude in China. Front Endocrinol (Lausanne). (2026) 17:1765770. 10.3389/fendo.2026.1765770 41710410 PMC12909196

[31] Pablos-Méndez A Mayeux R Ngai C Shea S Berglund L. Association of apo E polymorphism with plasma lipid levels in a multiethnic elderly population. Arterioscler Thromb Vasc Biol. (1997) 17(12):3534–41. 10.1161/01.ATV.17.12.3534 9437203

[32] Sniderman AD Thanassoulis G Glavinovic T Navar AM Pencina M Catapano A. Apolipoprotein B particles and cardiovascular disease: a narrative review. JAMA Cardiol. (2019) 4(12):1287–95. 10.1001/jamacardio.2019.3780 31642874 PMC7369156

[33] Zhang Y Li S Xu S Zhao R Zheng F Wu J. Apolipoprotein variations across APOE genotypes in young and elderly patients with coronary heart disease. Biosci Rep. (2026) 46(6):BSR20260258. 10.1042/BSR20260258 42206501 PMC13270160

[34] Kronenberg F Mora S Stroes ESG Ference BA Arsenault BJ Berglund L. Lipoprotein(a) in atherosclerotic cardiovascular disease and aortic stenosis: a European atherosclerosis society consensus statement. Eur Heart J. (2022) 43(39):3925–46. 10.1093/eurheartj/ehac361 36036785 PMC9639807

[35] Chen W Li B Wang H Wei G Chen K Wang W. Apolipoprotein E E3/E4 genotype is associated with an increased risk of type 2 diabetes mellitus complicated with coronary artery disease. BMC Cardiovasc Disord. (2024) 24(1):160. 10.1186/s12872-024-03831-0 38491412 PMC10941446

[36] Liu Q Wu H Yu Z Huang Q Zhong Z. APOE gene ε4 allele (388C-526C) effects on serum lipids and risk of coronary artery disease in southern Chinese Hakka population. J Clin Lab Anal. (2021) 35(9):e23925. 10.1002/jcla.23925 34313350 PMC8418481

[37] Karahan Z Uğurlu M Uçaman B Uluğ AV Kaya İ Çevik K. Relation between apolipoprotein E gene polymorphism and severity of coronary artery disease in acute myocardial infarction. Cardiol Res Pract. (2015) 2015:1–4. 10.1155/2015/363458 PMC4561336 26380146

[38] Li Y Zhong W Huang C Peng J Li H. Apolipoprotein E E3/E4 genotype is associated with an increased risk of premature coronary artery disease. BMC Cardiovasc Disord. (2024) 24(1):353. 10.1186/s12872-024-04021-8 38987708 PMC11234594

[39] Wang H Li B Chen W Wei G Chen K Wang W. ε3/ε4 genotype is associated with premature myocardial infarction: a hospital based retrospective study. BMC Cardiovasc Disord. (2026) 26(1):135. 10.1186/s12872-026-05513-5 41540357 PMC12892622

[40] Wei G Li B Wang H Chen L Chen W Chen K. Apolipoprotein E E3/E4 genotype is associated with an increased risk of coronary atherosclerosis in patients with hypertension. BMC Cardiovasc Disord. (2024) 24(1):486. 10.1186/s12872-024-04169-3 39261765 PMC11391850

[41] Karjalainen J-P Mononen N Hutri-Kähönen N Lehtimäki M Hilvo M Kauhanen D. New evidence from plasma ceramides links apoE polymorphism to greater risk of coronary artery disease in Finnish adults. J Lipid Res. (2019) 60(9):1622–9. 10.1194/jlr.M092809 31270131 PMC6718445

[42] Afroze D Yousuf A Tramboo NA Shah ZA Ahmad A. Apoe gene polymorphism and its relationship with coronary artery disease in ethnic Kashmiri population. Clin Exp Med. (2016) 16(4):551–6. 10.1007/s10238-015-0389-7 26409839

[43] Ma W Ren X Zhang L Dong H Lu X Feng W. Apolipoprotein E gene polymorphism and coronary artery disease risk among patients in northwest China. Pharmgenomics Pers Med. (2021) 14:1591–9. 10.2147/PGPM.S338285 34908864 PMC8665779

[44] Balcerzyk A Zak I Krauze J. Synergistic effects of apolipoprotein E gene epsilon polymorphism and some conventional risk factors on premature ischaemic heart disease development. Kardiol Pol. (2007) 65(9):1058–65. 17975753

[45] Luo JQ Ren H Banh HL Liu MZ Xu P Fang PF. The associations between apolipoprotein E gene epsilon2/epsilon3/epsilon4 polymorphisms and the risk of coronary artery disease in patients with type 2 diabetes mellitus. Front Physiol. (2017) 8:1031. 10.3389/fphys.2017.01031 29311965 PMC5732920

[46] Long Y Zhao XT Liu C Sun YY Ma YT Liu XY. A case-control study of the association of the polymorphisms of MTHFR and APOE with risk factors and the severity of coronary artery disease. Cardiology. (2019) 142(3):149–57. 10.1159/000499866 31163415

[47] Wang XL McCredie RM Wilcken DE. Polymorphisms of the apolipoprotein E gene and severity of coronary artery disease defined by angiography. Arterioscler Thromb Vasc Biol. (1995) 15:1030–4. 10.1161/01.ATV.15.8.1030 7627692

[48] Pitsavos C Choumerianou DM Skoumas J Maumus S Stefanadis C Dedoussis* GVZ. Apolipoprotein E polymorphism is not associated with lipid levels and coronary artery disease in Greek patients with familial hypercholesterolaemia. Clin Exp Med. (2005) 5(4):196–201. 10.1007/s10238-005-0086-z 16362800

[49] Bos MM De Vries L Rensen PCN Willems van Dijk K Blauw GJ Van Heemst D. Apolipoprotein E genotype, lifestyle and coronary artery disease: gene-environment interaction analyses in the UK biobank population. Atherosclerosis. (2021) 328:33–7. 10.1016/j.atherosclerosis.2021.05.014 34082327

[50] Poorzand H Tsarouhas K Hozhabrossadati SA Khorrampazhouh N Bondarsahebi Y Bacopoulou F. Risk factors of premature coronary artery disease in Iran: a systematic review and meta-analysis. Eur J Clin Invest. (2019) 49(7):e13124. 10.1111/eci.13124 31038733

[51] Khoja A Andraweera PH Lassi ZS Padhani ZA Ali A Zheng M. Modifiable and non-modifiable risk factors for premature coronary heart disease: systematic review and meta-analysis. Heart Lung Circ. (2024) 33(3):265–80. 10.1016/j.hlc.2023.12.012 38365496

[52] Sharma SK Makkar JS Bana A Sharma K Kasliwal A Sidana SK. Premature coronary artery disease, risk factors, clinical presentation, angiography and interventions: hospital-based registry. Indian Heart J. (2022) 74(5):391–7. 10.1016/j.ihj.2022.08.003 35995321 PMC9647652

[53] Le A Peng H Golinsky D Di Scipio M Lali R Paré G. What causes premature coronary artery disease? Curr Atheroscler Rep. (2024) 26(6):189–203. 10.1007/s11883-024-01200-y 38573470

[54] Schmitz F Mevissen V Krantz C Kimmel M Erdmann J Hoffmann R. Robust association of the APOE epsilon4 allele with premature myocardial infarction especially in patients without hypercholesterolaemia: the aachen study. Eur J Clin Invest. (2007) 37(2):106–8. 10.1111/j.1365-2362.2007.01764.x 17217375

[55] Shao A Shi J Liang Z Pan L Zhu W Liu S. Meta-analysis of the association between apolipoprotein E polymorphism and risks of myocardial infarction. BMC Cardiovasc Disord. (2022) 22(1):126. 10.1186/s12872-022-02566-0 35331149 PMC8952226

[56] Bouchareychas L Raffai RL. Apolipoprotein E and atherosclerosis: from lipoprotein metabolism to microRNA control of inflammation. J Cardiovasc Dev Dis. (2018) 5(2):30. 10.3390/jcdd5020030 29789495 PMC6023389

[57] Igel E Haller A Wolfkiel PR Orr-Asman M Jaeschke A Hui DY. Distinct pro-inflammatory properties of myeloid cell-derived apolipoprotein E2 and E4 in atherosclerosis promotion. J Biol Chem. (2021) 297(3):101106. 10.1016/j.jbc.2021.101106 34425108 PMC8437825

[58] Alagarsamy J Jaeschke A Hui DY. Apolipoprotein E in cardiometabolic and neurological health and diseases. Int J Mol Sci. (2022) 23(17):9892. 10.3390/ijms23179892 36077289 PMC9456500

[59] Mennander A Kuukasjärvi P Laurikka J Nikus K Karhunen PJ Tarkka M. Diagnostic performance of plasma high sensitive C-reactive protein in detecting three-vessel coronary artery disease: modification by apolipoprotein E genotype. Scand J Clin Lab Invest. (2008) 68(8):714–9. 10.1080/00365510802172145 18609102

[60] Larkin L Khachigian LM Jessup W. Regulation of apolipoprotein E production in macrophages. Int J Mol Med. (2000) 6(3):253–8. 10.3892/ijmm.6.3.253 10934285

[61] Yvan-Charvet L Wang N Tall AR. Role of HDL, ABCA1, and ABCG1 transporters in cholesterol efflux and immune responses. Arterioscler Thromb Vasc Biol. (2010) 30(2):139–43. 10.1161/ATVBAHA.108.179283 19797709 PMC2812788

[62] Heeren J Grewal T Laatsch A Becker N Rinninger F Rye K-A. Impaired recycling of apolipoprotein E4 is associated with intracellular cholesterol accumulation. J Biol Chem. (2004) 279(53):55483–92. 10.1074/jbc.M409324200 15485881

[63] Jofre-Monseny L Minihane AM Rimbach G. Impact of ApoE genotype on oxidative stress, inflammation and disease risk. Mol Nutr Food Res. (2008) 52(1):131–45. 10.1002/mnfr.200700322 18203129

[64] Vogel T Guo N-H Guy R Drezlich N Krutzsch HC Blake DA. Apolipoprotein E: a potent inhibitor of endothelial and tumor cell proliferation. J Cell Biochem. (1994) 54(3):299–308. 10.1002/jcb.240540306 8200910

[65] Kojima Y Weissman IL Leeper NJ. The role of efferocytosis in atherosclerosis. Circulation. (2017) 135(5):476–89. 10.1161/CIRCULATIONAHA.116.025684 28137963 PMC5302553

[66] Zhang L He S Li Z Gan X Li S Cheng X. Apolipoprotein E polymorphisms contribute to statin response in Chinese ASCVD patients with dyslipidemia. Lipids Health Dis. (2019) 18:129. 10.1186/s12944-019-1069-5 31153375 PMC6545221

[67] Chiodini BD Franzosi MG Barlera S Signorini S Lewis CM D'Orazio A. Apolipoprotein E polymorphisms and outcome in patients with coronary artery disease treated with statins: the GISSI-prevenzione study. Eur Heart J. (2007) 28(18):1977–83. 10.1093/eurheartj/ehm196 17567623

[68] Yoon YH Park GM Lee JY Lee JH Lee H Roh JH. Association of stage 1 hypertension defined by the ACC/AHA 2017 guideline with asymptomatic coronary atherosclerosis. Am J Hypertens. (2021) 34(8):858–66. 10.1093/ajh/hpab051 33735371

[69] Park HW Jo S Park KS Lee H Jeon YJ Park S. Differential impact of degree of hypertension on subclinical coronary atherosclerosis in asymptomatic subjects with and without diabetes mellitus. Am J Cardiol. (2023) 203:343–51. 10.1016/j.amjcard.2023.07.036 37517130

[70] Gallo G Volpe M Savoia C. Endothelial dysfunction in hypertension: current concepts and clinical implications. Front Med. (2022) 8:798958. 10.3389/fmed.2021.798958 PMC8811286 35127755

[71] Kondo T Nakano Y Adachi S Murohara T. Effects of tobacco smoking on cardiovascular disease. Circ J. (2019) 83(10):1980–5. 10.1253/circj.CJ-19-0323 31462607

[72] Teo KK Ounpuu S Hawken S Pandey MR Valentin V Hunt D. Tobacco use and risk of myocardial infarction in 52 countries in the INTERHEART study: a case-control study. Lancet. (2006) 368(9536):647–58. 10.1016/S0140-6736(06)69249-0 16920470

[73] Barua RS Ambrose JA. Mechanisms of coronary thrombosis in cigarette smoke exposure. Arterioscler Thromb Vasc Biol. (2013) 33(7):1460–7. 10.1161/ATVBAHA.112.300154 23685556

[74] Powell-Wiley TM Poirier P Burke LE Després J-P Gordon-Larsen P Lavie CJ. Obesity and cardiovascular disease: a scientific statement from the American Heart Association. Circulation. (2021) 143(21):e984–e1010. 10.1161/CIR.0000000000000973 33882682 PMC8493650

[75] Formentini FS Nagano FEZ Lopez Neto FDN Adam EL Fortes FS Silva LFD. Coronary artery disease and body mass index: what is the relationship? Clin Nutr ESPEN. (2019) 34:87–93. 10.1016/j.clnesp.2019.08.008 31677718

[76] Wang K Shi X Zhu Z Hao X Chen L Cheng S. Mendelian randomization analysis of 37 clinical factors and coronary artery disease in east Asian and European populations. Genome Med. (2022) 14(1):63. 10.1186/s13073-022-01067-1 35698167 PMC9195360

[77] Cavallari I Cannon CP Braunwald E Goodrich EL Im KA Lukas MA. Metabolic syndrome and the risk of adverse cardiovascular events after an acute coronary syndrome. Eur J Prev Cardiol. (2018) 25(8):830–8. 10.1177/2047487318763897 29537291

[78] Liehn EA Lupan AM Diaconu R Ioana M Streata I Manole C. Heart function assessment during aging in apolipoprotein E knock-out mice. Discoveries (Craiova). (2021) 9(3):e136. 10.15190/d.2021.15 34816004 PMC8605688

[79] Diaconu R Schaaps N Afify M Boor P Cornelissen A Florescu RA. Apolipoprotein E4 is associated with right ventricular dysfunction in dilated cardiomyopathy—an animal and in-human comparative study. Int J Mol Sci. (2021) 22(18):9688. 10.3390/ijms22189688 34575848 PMC8468742

