# Supplementary material for: Apolipoprotein E ε3/ε4 and coronary artery disease: lipid and non-lipid mechanisms in personalized prevention — narrative review
Source: Front Cardiovasc Med. 2026 Sep 18;13:1885898. doi: 10.3389/fcvm.2026.1885898 (PMC13631420; doi:10.3389/fcvm.2026.1885898)
Supplement: Supplementary file 1 [file Datasheet1.docx]

**SUPPLEMENTARY MATERIAL**

**Apolipoprotein E ε3/ε4 and Coronary Artery Disease: Lipid and Non-Lipid Mechanisms in Personalized Prevention — Narrative Review**

*Supplementary Table S1. Database-specific search strategies*

# 1. Overview

A single search strategy was run in PubMed, Embase, Scopus, and Web of Science from database inception to August 2026. The strategy combines an exposure block (APOE gene and protein terms) with a topic block (coronary artery disease, premature coronary artery disease, atherosclerosis, lipid metabolism, cardiovascular genetics, and personalized prevention), joined with the Boolean operator AND. The syntax below is reproduced as executed in each interface, so that the retrieval can be reproduced by any reader. Results from the four databases were combined and deduplicated before screening.

| **Database** | **Interface / field codes** | **Limits applied** |
| --- | --- | --- |
| **PubMed (MEDLINE)** | NCBI; MeSH, [tiab] | English language |
| **Embase** | Elsevier; Emtree /exp, :ti,ab | [english]/lim |
| **Scopus** | Elsevier; TITLE-ABS-KEY | LIMIT-TO (LANGUAGE, “English”) |
| **Web of Science** | Clarivate; TS = Topic | Languages: English |

# 2. Search strategy

## 2.1 PubMed (MEDLINE)

```
("Apolipoprotein E"[MeSH Terms] OR "apolipoprotein E"[tiab] OR "APOE"[tiab]
 OR "APOE polymorphism"[tiab] OR "APOE e3/e4"[tiab]
 OR "APOE epsilon 3/epsilon 4"[tiab])
AND
("Coronary Artery Disease"[MeSH Terms] OR "coronary artery disease"[tiab]
 OR "premature coronary artery disease"[tiab]
 OR "Atherosclerosis"[MeSH Terms] OR "atherosclerosis"[tiab]
 OR "Lipid Metabolism"[MeSH Terms] OR "lipid metabolism"[tiab]
 OR "cardiovascular genetics"[tiab] OR "personalized prevention"[tiab])
Filters: English
```

## 2.2 Embase

```
('apolipoprotein E'/exp OR 'apolipoprotein e':ti,ab OR apoe:ti,ab
 OR 'apoe polymorphism':ti,ab OR 'apoe e3/e4':ti,ab)
AND
('coronary artery disease'/exp OR 'coronary artery disease':ti,ab
 OR 'premature coronary artery disease':ti,ab
 OR 'atherosclerosis'/exp OR atherosclerosis:ti,ab
 OR 'lipid metabolism'/exp OR 'lipid metabolism':ti,ab
 OR 'cardiovascular genetics':ti,ab OR 'personalized prevention':ti,ab)
AND [english]/lim
```

## 2.3 Scopus

```
TITLE-ABS-KEY(("apolipoprotein E" OR APOE OR "APOE polymorphism"
 OR "APOE e3/e4")
 AND ("coronary artery disease" OR "premature coronary artery disease"
 OR atherosclerosis OR "lipid metabolism" OR "cardiovascular genetics"
 OR "personalized prevention"))
AND (LIMIT-TO (LANGUAGE, "English"))
```

## 2.4 Web of Science

```
TS=(("apolipoprotein E" OR APOE OR "APOE polymorphism" OR "APOE e3/e4")
 AND ("coronary artery disease" OR "premature coronary artery disease"
 OR atherosclerosis OR "lipid metabolism" OR "cardiovascular genetics"
 OR "personalized prevention"))
Refined by: Languages = English
```

# 3. Eligibility criteria

|  | **Criteria** |
| --- | --- |
| **Included** | English-language publications; original research articles, narrative reviews, and meta-analyses; reporting on associations between APOE genotype — with specific emphasis on the ε3/ε4 genotype — and CAD, premature CAD, lipid metabolism, or ApoE-mediated biological mechanisms. |
| **Excluded** | Studies with fewer than five participants; conference abstracts without full text; publications focused exclusively on APOE in neurodegenerative disease without cardiovascular outcomes. |

# 4. Additional sources

Reference lists of all retrieved full-text articles and of previous reviews on APOE and cardiovascular disease were hand-searched for publications not identified by the electronic search. This route accounts for most of the primary experimental sources cited in support of the non-lipid mechanisms discussed in Section 7 of the main text, which were not the target of the electronic strategy. Grey literature, trial registries, and preprint servers were not searched.

# 5. Note on review design

This review was conducted as a narrative synthesis. Record counts, duplicate-removal figures, and a PRISMA flow diagram are therefore not reported, and no protocol was registered. The rationale is given in Section 2.1 of the main text, and reporting follows SANRA, the validated instrument for non-systematic reviews. The search strategies above are provided so that the retrieval underlying the synthesis is fully reproducible.
